# Seasonal dynamics and core stability of the bacterial microbiome of a *Drosophila suzukii* wild population

**DOI:** 10.1038/s41598-026-37656-y

**Published:** 2026-01-29

**Authors:** Marino Costa-Santos, Sara Sario, Rafael J. Mendes, Conceição Santos

**Affiliations:** 1 Biology Department, Faculty of Science (FCUP), University of Porto, 4169-007 Porto, Portugal; 2 LAQV-REQUIMTE, Biology Department, Faculty of Science (FCUP), University of Porto, 4169-007 Porto, Portugal

**Keywords:** Spotted-wing drosophila, Summer and winter morphotypes, Bacteriota, Core microbiome, Microbiome shifts, Host-microbe interaction, Ecology, Ecology, Microbiology, Zoology

## Abstract

*Drosophila suzukii* (spotted-wing drosophila, SWD) is an invasive pest with pronounced sexual dimorphism and seasonal polyphenism. While seasonal morphotypes are well documented, how these phenotypic traits shape the SWD microbiome remains poorly understood. Here, we investigate how sex and seasonal phenotypes shape microbiome composition in SWD. We hypothesize that these factors drive microbial shifts, with some taxa varying between phenotypes and others forming a stable core. Understanding these patterns may reveal microbiome-associated adaptations relevant to SWD ecology and management. To investigate this, we monitored SWD microbiome dynamics over one year by collecting individuals during spring, summer, and autumn of 2022 and winter of 2023 from an organic farm in northern Portugal. Bacterial communities were compared using 16 S rRNA amplicon sequencing. This SWD population retained a core bacterial community, highly represented by *Gluconobacter*, *Pseudomonas*, *Commensalibacter* and *Pantoea*, consistent with other SWD Portuguese populations. Moreover, microbiome composition varied significantly across seasons but not between sexes, although females exhibited higher microbial alpha diversity. Linear discriminant analysis of relative abundance (LEfSe) revealed enrichment of *Morganella*, *Methanosaeta*, *Serratia*, *Duganella*, *Frateuria*, *Suttonella*, and *Janthinobacterium* in winter groups. However, functional prediction analyses revealed no significant differences in microbiome functional potential across seasons, suggesting functional redundancy despite taxonomic variation. This study offers baseline insights into the seasonal stability and plasticity of the *D. suzukii* microbiome, contributing to a deeper ecological understanding of this invasive pest.

**Supplementary Information:**

The online version contains supplementary material available at 10.1038/s41598-026-37656-y.

## Introduction

*Drosophila suzukii* (SWD) remains the most relevant pest of soft-skin fruits and berries. This sexually dimorphic pest is highly polyphagous, and its females have a distinctive serrated oviscape, which allows SWD to explore niches unavailable to other *Drosophila* species. Also, its larvae grow inside the ripening fruits, protected from insecticides^1,2^. The lack of effective control measures and the low market tolerance for infested fruits lead to economic losses^3,4^.

Native from Southeast Asia, SWD established itself around the last decade in the Mediterranean region, enabled by climate change and its ability to tolerate the cold winters^5^. SWD winter survival is mainly associated with its seasonal polyphenism, as well as adaptations in diet, mating, and dispersal behaviors^6^. Although winter morphs and summer morphs physiological and phenotypic differences are relatively well known, with winter traits such as increased body melanization and wing length supporting pest survival during winter^7^, the dynamics of SWD microbiome along seasons are less studied.

The SWD microbial communities include surface, intracellular and, mostly, gut microbes, and include both core and transient taxa, dependent on season and other environmental factors^8,9^. Like in all microbiomes, SWD’s microorganisms establish different metabolic networks between each other and with the host. These networks range from detrimental to beneficial^10^. For example, beneficial gut microorganisms maintain epithelium integrity, help with nutritional requirements, provide protection against pathogenic microorganisms, and/or regulate host immunity^11–14^. Some microorganisms may also influence social interactions like aggressive behaviors, and preferences for mating or oviposition^15,16^. Pathogenic ones can act by producing toxins^17^ or disrupting host’s homeostasis^18^.

A few studies propose that some microorganisms might be persistently associated with SWD, such as members of the Enterobacteriacea, Lactobacillaceae and Acetobacteriaceae families^8,10,19^ and yeasts such as *Candida* spp. and *Saccharomyces* spp^19–21^. Persistent bacteria may also prevail over fungal associations with SWD^22^, but the identification of a core microbiome in SWD remains unclear. Several authors converge on showing that field microbiomes are particularly dynamic, depending on intrinsic and environmental factors. In particular, some taxa may be constantly replenished through feeding from fruits, flowers or water reservoirs^23–25^.

Environmental changes lead to shifts in diet and behavior and, thus, can alter the microbiomes. For instance, olfactory preferences shift within seasons^26^, and alternative diets such as decomposing organic matter^27^ or possibly bird manure^28^ may also impact *Drosophila*’s microbiota structure. Also, the microbiota of ectothermic animals, like insects, might be linked to cold tolerance and immunity^29^. Studies in *Drosophila* spp. suggest that microbiomes might play a role in cold-stress responses^30–32^, in a way yet to be fully elucidated. However, there are not many studies focusing on the changes of SWD microbiomes in function of season and sex.

Here, it is hypothesized that adult SWD has a prevalent bacterial community that is constituted by “core” taxa, less susceptible to polyphenisms, alongside transient taxa dependent on environmental conditions and sex. These shifts in microbiome composition might support adaptation to suboptimal environments, e.g. winter-enriched taxa could be involved in increased tolerance to cold and compensation for nutritionally limited diets.

In this study, we profiled the bacterial communities of a Portuguese *Drosophila suzukii* population across spring, summer, autumn, and winter using 16 S rRNA amplicon sequencing, to identify resident taxa and evaluate seasonal and sex-associated shifts in community structure and functional potential.

## Methods

### SWD sampling

Wild SWD individuals were captured during 2022 and 2023 in Santa Maria da Feira (SMF), Aveiro, Portugal, in a biological production (without the application of agrochemicals) containing infested raspberries and grapes. This production was monitored approximately every 60 days, resulting in the capture of summer morphotype individuals in Spring (early June 2022), Summer (late August 2022), Autumn (late October 2022), and winter morphotype individuals in Winter (early January 2023). Field sampling was done resorting to 10 Drososan^®^ traps baited with fruit fly attractant (Koppert, Almería, Spain) distributed randomly across the production near the fruits, under shading if possible, and hanging on the same spots for all collection time points. Permission to conduct field sampling was obtained from the landowner of the production site prior to the deployment of traps and collection of flies. The lure was discarded after each use. Flies were collected from the traps after a maximum of 8 h of exposure to minimize possible feeding from the lure, and transported in sterile 50 mL tubes, in ice. Flies were immediately processed after each capture or stored at −80 °C until used. Identification of captured summer and winter morphotypes was done according to recent morphometric criteria^33^.

In addition to the main SMF population, sampling was performed in 4 other regions from the North of Portugal for comparative purposes, namely Oliveira de Azeméis in a Persimmon orchard (Summer/Autumn, 2022), Vila Nova de Gaia in a Blueberry production farm (Summer, 2022), Póvoa de Varzim in a Raspberry production farm (Summer, 2022) and Castelo de Paiva for Raspberry (Summer, 2022).

### Total DNA extraction

Captured individuals were surface disinfected with bleach (1% v/v) for 10 min and washed three times with sterile dH_2_O. Individuals were then separated by sex and pooled in groups of 3 to 5 flies, depending on the number of captures, resulting in 3 pools of males and 3 pools of females for each condition. Pools were processed on a Fisherbrand™ Bead Mill 24 Homogenizer (Fisher Scientific, Waltham, MA, USA) at 6 m/s for 10 s, using sterilized homogenization tubes and 2 mm diameter ceramic beads. Total DNA was extracted using the QIAamp^®^ DNA Micro Kit (QIAGEN^®^, Hilden, Germany), following the Protocol for Isolation of Genomic DNA from Tissues according to the manufacturer’s instructions. DNA purity and concentration were evaluated using the LVis plate on a FLUOstar Omega Microplate Reader (BMG Labtech, Ortenberg, Germany), following the manufacturer’s protocols.

### 16 S metagenomic sequencing and analysis

Amplicon 16 S rRNA metagenomic sequencing was outsourced to Novogene Europe (Novogene Co., Cambridge, UK). Illumina libraries were generated after PCR amplification of the V3-V4 hypervariable regions of 16 S rRNA gene. Samples were sequenced in the Illumina NovaSeq 6000 platform. The raw 16 S rRNA gene sequencing data were processed using QIIME2 for quality control, feature table construction, and taxonomic classification. After demultiplexing, sequences were denoised using the DADA2 plugin to generate amplicon sequence variants (ASVs). Taxonomic assignment was performed against the SILVA database^34^.

The resulting feature table (after removing chloroplast and mitochondria ASVs), taxonomy data, and sample metadata file were uploaded to the MicrobiomeAnalyst web server (https://www.microbiomeanalyst.ca) for comprehensive downstream analysis and visualization^35,36^. Within the platform, data were filtered to remove low-count and low-variance features and normalized via Total Sum Scaling (TSS), using default settings. Alpha and Beta diversity analyses were performed at the ASV level. ASVs were not collapsed by taxonomic assignment, retaining the resolution provided by DADA2 inference. For compositional summaries (e.g., barplots), ASVs were collapsed to the genus level to facilitate interpretation. Alpha diversity metrics (Chao1 and Shannon indices; analyzed through Kruskal-Wallis or Mann-Whitney U test) and beta diversity analysis (Principal Coordinate Analysis based on Bray–Curtis dissimilarities; Differences were assessed using PERMANOVA) were calculated. Taxonomic composition was visualized through pie charts (phylum and genus levels) and bar plots (genus level). Additional visualizations included clustering-based dendogram and heatmap, as well as core microbiome heatmaps. Differential abundance analysis was conducted using the Linear Discriminant Analysis Effect Size (LEfSe) method to identify taxa with significant variation across groups (P value < 0.1 and LDA score > 2).

### Functional profiling and statistical analyses

Microbial functional potential was predicted from 16 S rRNA gene data using PICRUSt2 (v2.5.2), run on a macOS environment via the command line, using default parameters^37^. The predicted MetaCyc pathway abundances and KEGG Orthologs (KOs)^38–40^ were imported into R (v4.4.0) for downstream visualization and statistical analysis.

For multivariate analysis, the MetaCyc pathway matrix and the KO matrix were transposed and scaled, then analyzed using Principal Component Analysis (PCA) via the prcomp() function in base R. Group differences were assessed using PERMANOVA (adonis function, vegan package (v2.6.10)^41^ based on Bray-Curtis dissimilarities to quantify the effect of Season and Sex on predicted functional profiles. For heatmaps, feature abundances were were standardized (z-scored by row) and visualized as a heatmap using the pheatmap package, with samples annotated by season.

In parallel, Clusters of Orthologous Groups (COG)-based functional profilling was performed using the MicrobiomeAnalyst web platform (https://www.microbiomeanalyst.ca^35,36^;, based on taxonomic profiles classified against the SILVA database^34^.

## Results

### 16 S metagenomic sequencing

Sequencing of 16 S V3-V4 amplicons produced 2,960,579 raw paired-end reads that, after quality check and removal of chimeric sequences, produced 2,338,452 effective tags (79.03%). Of these, 11,318 ASVs were identified with an average length of 420 nt and a 98.23% Q20 score. Sequencing data and quality are summarized in **Supplementary Table ****S1**.

### Alpha diversity analysis

**Fig. 1:**
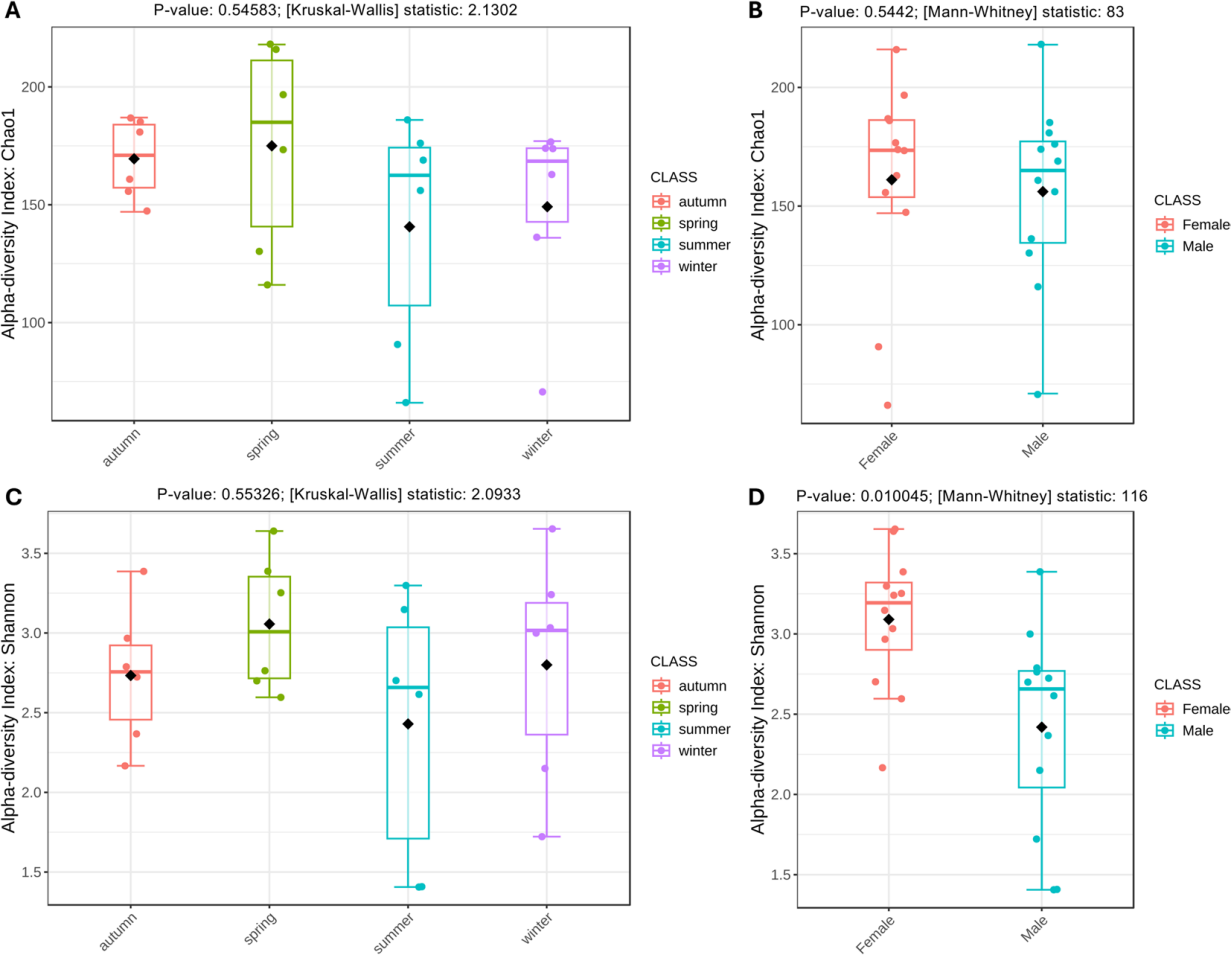
ASV-level Alpha diversity metrics (Chao1 richness index and Shannon diversity index) of microbial communities across samples. Horizontal lines represente the median, the black diamonds represente the mean, and the whiskers extend to 1.5 times the interquartile range from the box. (**A**) – Chao1 index across the grouping factor “Season”. Kruskal-Wallis test: P-value = 0.54583, Test statistic: 2.1302; (**B**) – Chao1 index across the grouping factor “Sex”. Mann-Whitney test: P-value = 0.5442, Test statistic: 83; (**C**) – Shannon index across the grouping factor “Season”. Kruskal-Wallis test: P-value = 0.55326, Test statistic: 2.0933; (**D**) – Shannon index across the grouping factor “Sex”. Mann-Whitney test: P-value = 0.010045, Test statistic: 116.

This analysis provides information on the diversity of the SWD population considering the season and sex groups. The Chao1 index (estimated number of ASVs) was used to assess community richness in female and male groups and in seasonal groups. No significant differences were observed across different seasons (P-value = 0.5442) (Fig. 1A), nor between males and females (P value = 0.5458) (Fig. 1B).

Similarly, no significant differences were observed when comparing seasonal groups for the Shannon index (P-value = 0.5533) (Fig. 1C). In contrast, the Shannon index (which measures richness and evenness) was significantly higher for female pools (P-value = 0.0100) (Fig. 1D), comparatively to male counterparts. These results indicate that female groups tend to have a richer and more even community comparatively to male groups, while seasonality didn’t seem to influence these metrics.

### Microbial community profiling

To have a general idea of the microbial communities harbored by SWD, relative aggregated abundances were plotted at the phylum and at the genus level. Then, samples were grouped by season and sex to build an abundance histogram, contemplating the 20 most abundance genera throughout SWD pools.

**Fig. 2:**
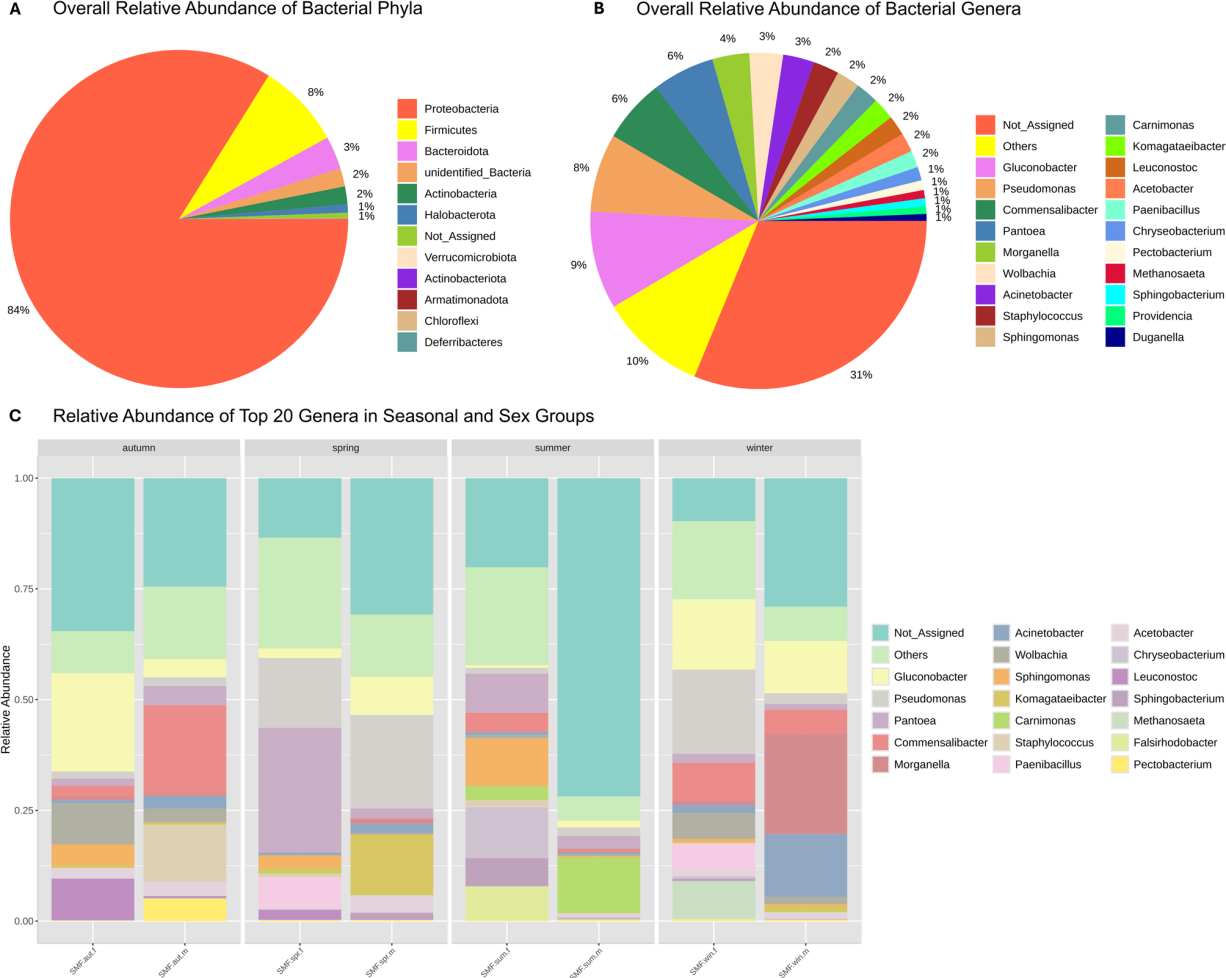
– Microbial community profiling. (**A**) Pie chart of the relative abundance throughout samples, at the phylum level. (**B**) Pie chart of the relative abundance throughout samples, at the genus level. (**C**) Histogram barplot of the top 20 genera abundances in both seasonal and sex groups.

SWD microbiomes were dominated by Proteobacteria (82%), followed by Firmicutes (8%), Bacteroidota (3%), unidentified bacteria (2%), Actinobacteria (2%) and Halobacterota (1%) (Fig. 2A). At the genus level *Gluconobacter* accounted for 9% of all observed genera, followed by *Pseudomonas* (8%), *Commensalibacter* (6%) and *Pantoea* (6%), *Morganella* (4%), the endosymbiont *Wolbachia* (3%), *Acinetobacter* (3%). Genera such as *Staphylococcus*, *Sphingomonas* and *Carnimonas*, *Komagataeibacter*, *Leuconostoc*, *Acetobacter* and *Paenibacillus* each represented 2% of all bacteria (Fig. 2B).

The histogram barplot (Fig. 2C) showed that Spring males bacterial communities were dominated by *Pseudomonas* (21,11%), followed by *Komagataeibacter* (13,47%), Gluconobacter (8.65%), *Acetobacter* (3,67%) and *Pantoea* (2,22%). Spring females’ communities were dominated by *Pantoea* (28,13%), followed by *Pseudomonas* (15,76%), *Sphingomonas* (2,75%), *Leuconostoc* (2,18%) and *Gluconobacter* (2,16%).

Summer males microbiome was dominated by *Carnimonas* (12,50%), followed by *Pantoea* (2,80%), *Pseudomonas* (1,90%), *Gluconobacter* (1,57%) and *Commensalibacter* (0,75%), while female counterparts’ bacterial communities were predominantly constituted by *Sphingomonas* (10,67%), *Pantoea* (8,80%), *Commensalibacter* (4,31%), *Carnimonas* (2,97%) and S*taphylococcus* (1,49%).

Autumn males main bacterial genera was *Commensalibacter*, representing 20,01% of all genera, followed by *Staphylococcus* (12,83%), *Pectobacterium* (5,04%), *Pantoea* (4,35%) and *Gluconobacter* (4,08%). The Acetobacteriaceae family was also highly prevalent in autumn females, with *Gluconobacter* accounting for 22,20% of all detected genera. *Wolbachia* accounted for 9,36% of all genera. *Leuconostoc*, *Sphingomonas* and *Commensalibacter* represented 9,33%, 4,65% and 2,46% of all bacteria, respectively.

The phylum Proteobacteria was strongly represented in winter males, being *Morganella* the dominant genus (22.50%), followed by *Acinetobacter* (14.15%), *Gluconobacter* (11,76%), *Commensalibacter* (5,40%) and *Pseudomonas* (2,46%). Winter females’ main bacteria were *Pseudomonas* (18.57%) and the acetobacteria *Gluconobacter* (15.56%), followed by *Commensalibacter* (8,37%) and *Pantoea* (2,05%). The endosymbiont *Wolbachia* represented 5,72% of all bacteria in winter females.

### Core genera

**Fig. 3:**
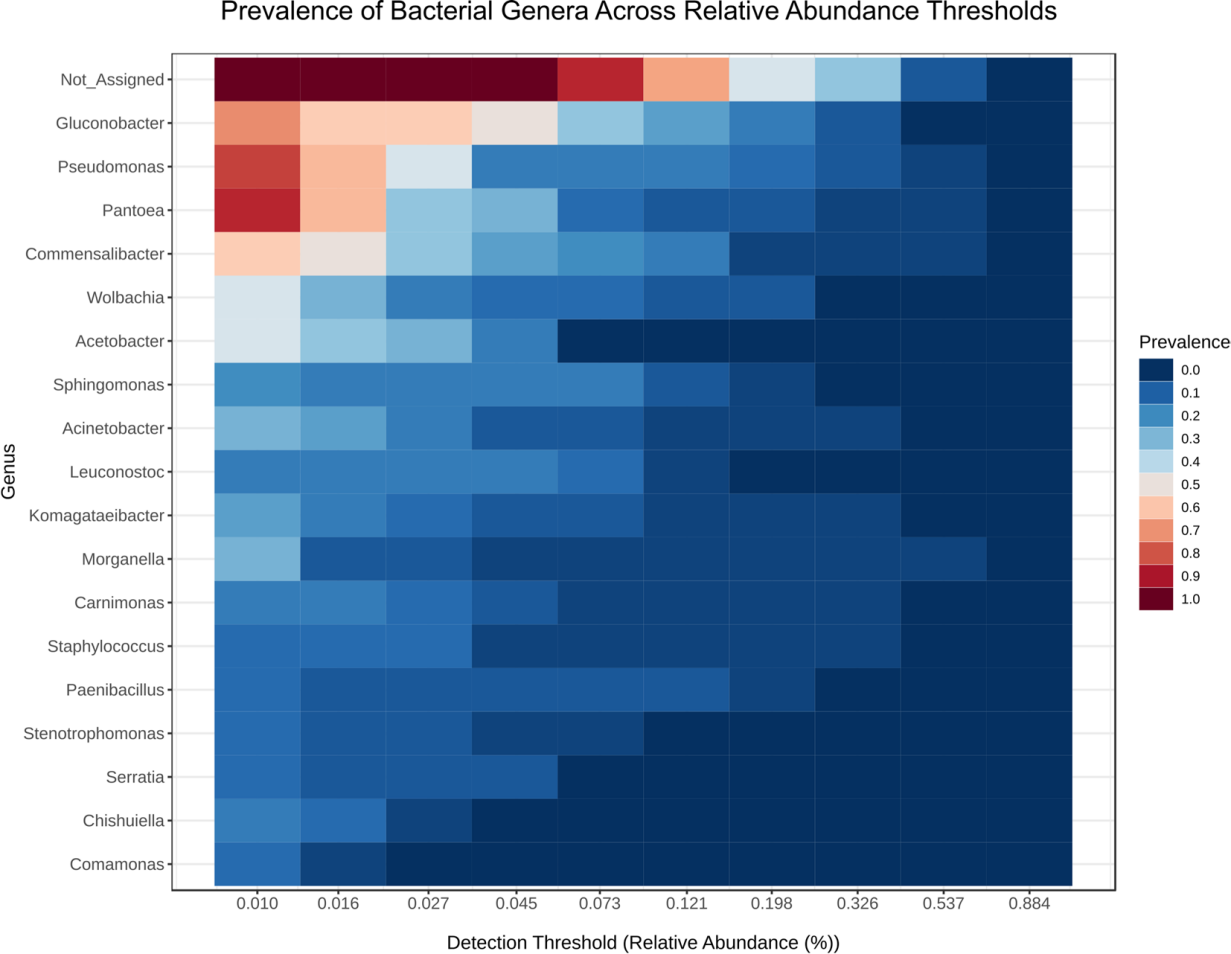
Prevalence of Bacterial Genera Across Different Relative Abundance Detection Thresholds. The heatmap illustrates the prevalence (proportion of samples in which a genus is detected) of the most abundant bacterial genera. Rows represent individual genera, and columns represent increasing detection thresholds for relative abundance (ranging from 1% to 88.4%). The color intensity indicates the prevalence of each genus at a given threshold, as per the color scale bar on the right (dark blue = 0% prevalence, dark red = 100% prevalence). Genera are sorted by decreasing overall abundance.

To identify probable core microbiome members, a heatmap of genus-level relative abundances across all SMF pools was generated (Fig. 3). No single genus was detected in every sample pool, although several taxa were consistently detected at notable abundances. *Pantoea* was the most prevalent, found in 90% of pools at ≥ 1% relative abundance. *Pseudomonas* and *Gluconobacter* were present in 80% and 70% of pools, respectively, also at ≥ 1%. Notably, *Gluconobacter* reached ≥ 4.5% abundance in 50% of samples. *Commensalibacter* was found in 60% of pools at ≥ 1%, while *Wolbachia* and *Acetobacter* each appeared in 50% of samples at the same threshold. Given their consistent presence (in ≥ 50% of all pools, regardless of sex or seasonal polyphenism, in an abundance of ≥ 1%) these genera can be considered part of the “core microbiome” of this SWD population.

Additionally, a heatmap including other Portuguese populations was constructed to further determine if these core genera are also present despite differences in region and crop (**Supplementary Figure I**). These additional populations were collected in 2022 in 4 other farms, also in the north of Portugal, namely in Oliveira de Azeméis (Raspberry in 2021 and Persimmon in 2022), Vila Nova de Gaia (Blueberry in 2022), Póvoa de Varzim (Raspberry in 2022) and Castelo de Paiva (Raspberry in 2022), sampled throughout the Spring and Summer. This sampling and analysis followed the same methodology as the SMF population.

The inclusion of these additional populations yielded similar results, with the heatmap revealing a set of recurrent genera across all five populations. *Gluconobacter* was the most prevalent, detected in 90% of all pools, followed by *Pantoea* (80%), *Pseudomonas* (70%), *Commensalibacter* and *Acetobacter* (60%), and *Wolbachia* (50%), all at ≥ 1% relative abundance. The consistent presence of these genera across geographically distinct populations suggests that they may constitute core members of the SWD microbiome, at least within this regional context.

### Beta diversity analysis

The PCoA analysis based on the Bray-Curtis dissimilarity distance matrix revealed that, generally, males and females of the same season harbored more closely related bacterial communities than those of different seasons.

**Fig. 4:**
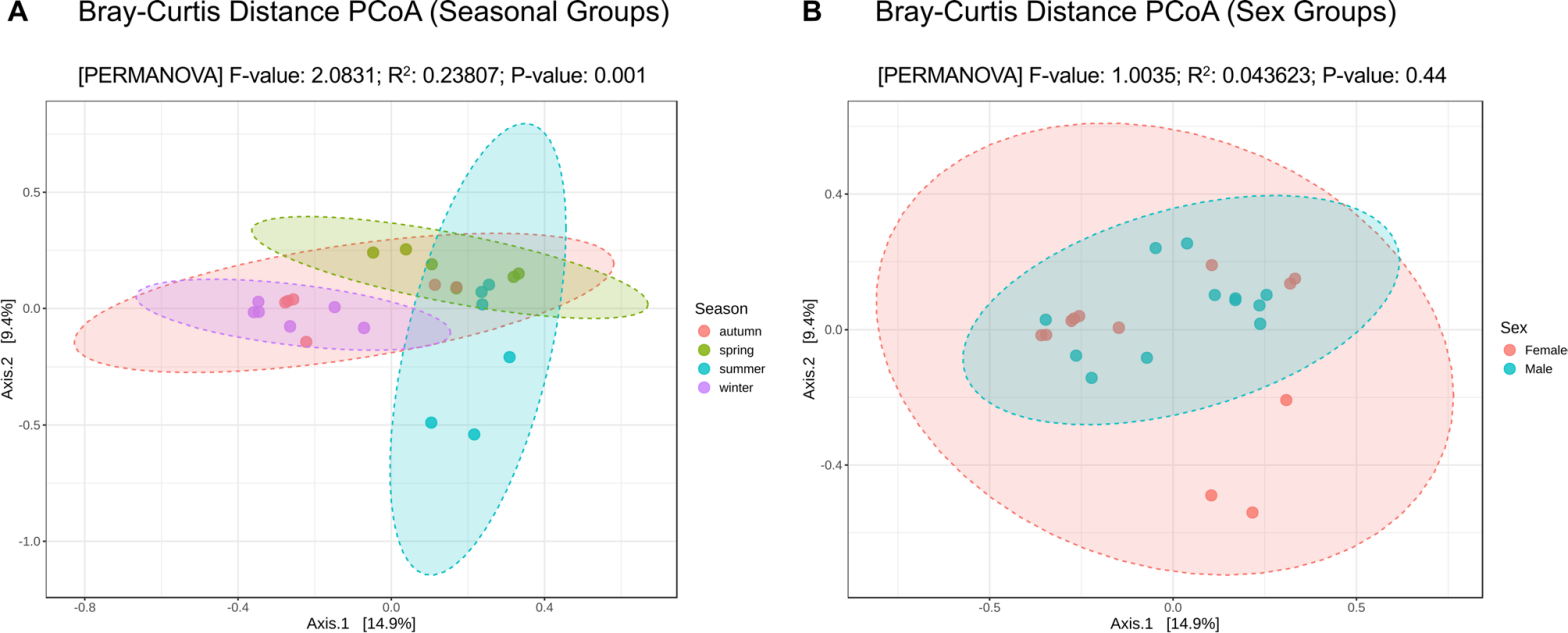
PCoA plots based on the Bray-Curtis dissimilarity matrix of different SWD groups, showing the first two principal coordinates, PCo1 and PCo2, that explain 14.9% and 9.4% of variance between groups. (**A**) PCoA plot on Seasonal groups. PERMANOVA: F-value: 2.0831; R^2^: 0.23807; P-value: 0.001. (**B**) PCoA plot on Sex groups. PERMANOVA: F-value: 1.0035; R^2^: 0.043623; P-value: 0.44.

Regarding season (Fig. 4A), summer and spring groups tend to cluster together, while the same applies to winter and autumn groups, although most of the variance remains unexplained, with PCo1 explaining 14.7% of total variance and PCo2 explaining 9.3% of total variance between samples. Seasonal differences contribute to a portion of the variation in microbiome composition, making this factor an important determinant, as supported by the PERMANOVA analysis. The F-value of 2.0831 indicates moderate separation among the groups based on seasonal differences; The R^2^ of 0.23807 suggests that 23.8% of the variation in microbiome composition can be explained by the seasonal factor, and the P-value of 0.001 indicates that the differences between the seasons are statistically significant.

Regarding sex (Fig. 4B), both the PCoA analysis and the PERMANOVA statistics suggest that this factor does not significantly explain differences in the microbiome composition of SWD individuals. The overlapping of the ellipses supports the lack of differences between groups. Moreover, the F-value of 1.0035 indicates that the variation between sexes is virtually identical to the random variation within them. Accordingly, the R^2^ of 0.043623 suggests that only 4.4% of the variation could be explained by the sex of the flies, and the P-value of 0.465, indicates that the observed differences between groups are not statistically significant.

### Community differences analysis

#### Clustering/Correlation analysis

**Fig. 5:**
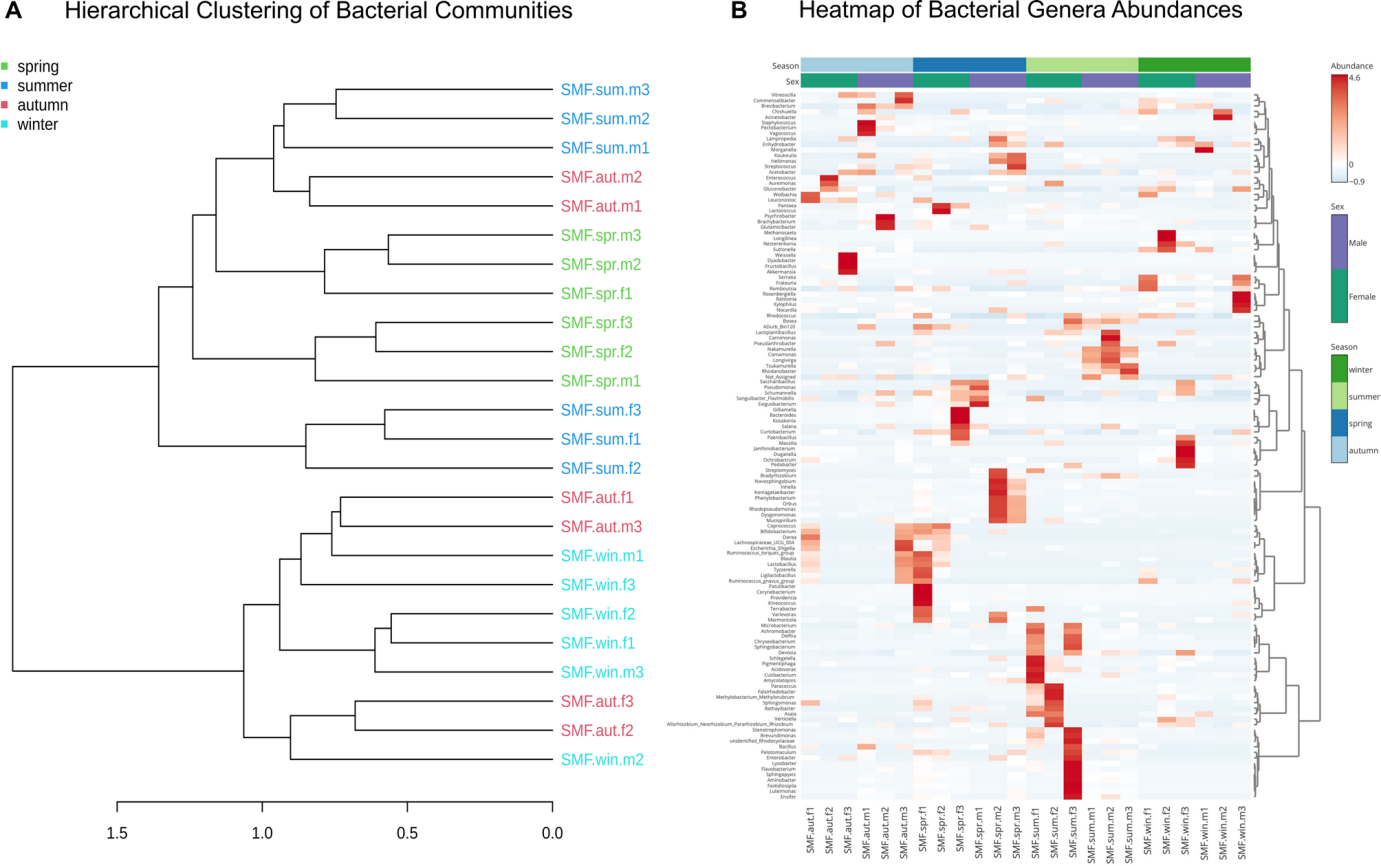
**(A)** Hierarchical clustering dendrogram of microbial communities based on Bray-Curtis distances. The dendrogram was constructed using Ward’s clustering method to group samples by community composition, with clustering performed at the ASV level. (**B**) Heatmap showing the relative abundances of 80 bacterial genera across sample, constructed using hierarchical clustering with Ward’s clustering method and Euclidean distance to group both samples and taxa. Color intensity represents the normalized abundance of taxa, with warmer colors indicating higher abundance.

The clustering dendrogram (Fig. 5A) of the microbial communities showed that all the winter samples branched together with all autumn females and one pool of autumn males, in a main independent branch. Summer and spring pools clustered on a different branch, together with the other two autumn male pools. This clustering pattern indicates that the cold seasons microbiomes are more related to each other than to the hot seasons microbiomes, and vice-versa. To further investigate correlation between groups, a heatmap at the genus level was constructed (Fig. 5B), which highlighted the most abundant genera within each pool of individuals. This analysis showed that, while the same sex and season SWD tend to share similar patterns in the heatmap, there is a high degree of variation among pools, indicating high plasticity of the microbiomes.

#### LEfSe (linear discriminant analysis (LDA) effect Size)

**Fig. 6:**
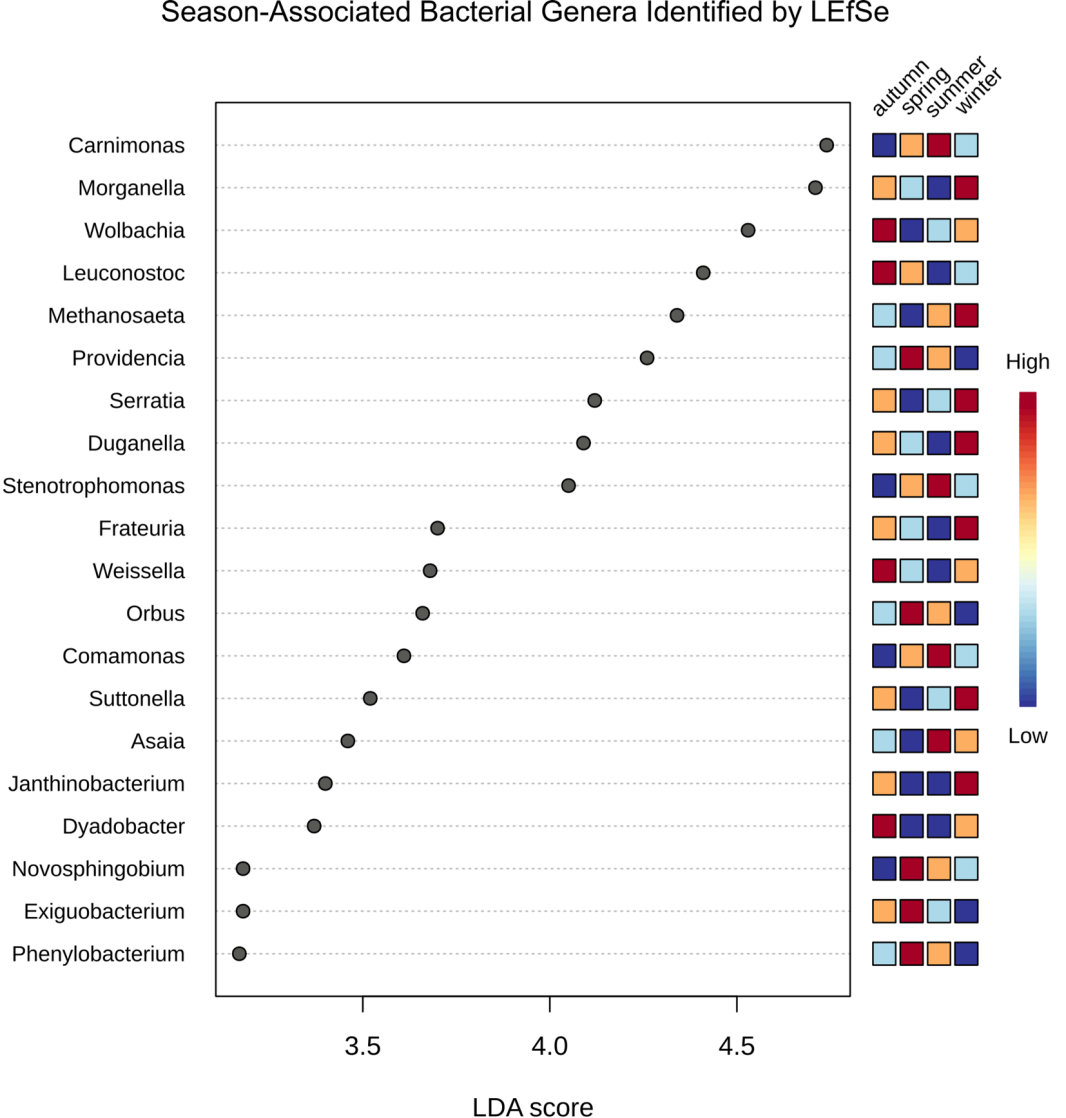
Linear Discriminant Analysis (LDA) effect size (LEfSe) dot plot highlighting the top 20 significantly enriched microbial taxa across seasons (Threshold: LDA > 2; P-value < 0.1). Each dot represents a microbial taxon, and the position indicates the LDA score, reflecting the effect size.

To further discriminate the differences at the seasonal level, LDA LEfSe analysis comparing all seasonal groups, at the genus resolution, with a threshold of P-value < 0.1 and LDA score > 2 was performed, aiming at identifying seasonally associated microbial biomarkers, and the top 20 genera were plotted. This analysis (Fig. 6) revealed that the spring group displayed higher abundances of *Providencia* (LDA score of 4.26, P-value = 0.0235), *Orbus* (LDA score of 3.66, P-value = 0.0010), *Novosphingobium* (LDA score of 3.18, P-value = 0.0132), *Exiguobacterium* (LDA score of 3.18, P-value = 0.0147) and *Phenylobacterium* (LDA score of 3.17, P-value = 0.0008), while the summer group had higher abundances of *Carnimonas* (LDA score of 4.74, P-value = 0.0080), *Stenotrophomonas* (LDA score of 4.05, P-value = 0.0107), *Comamonas* (LDA score of 3.61, P-value = 0.0159) and *Asaia* (LDA score of 3.46, P-value = 0.0235).

The autumn group presented higher abundances of the genera *Wolbachia* (LDA score of 4.53, P-value = 0.0196), *Leuconostoc* (LDA score of 4.41, P-value = 0.0095), *Weissela* (LDA score of 3.68, P-value = 0.0011) and *Dyadobacter* (LDA score of 3.37, P-value = 0.0043), while the winter group was distinguished by high abundances of *Morganella* (LDA score of 4.71, P-value = 0.0189), *Methanosaeta* (LDA score of 4.34, P-value = 0.0256), *Serratia* (LDA score of 4.12, P-value = 0.0055), *Duganella* (LDA score of 4.09, P-value = 0.0017), *Frateuria* (LDA score of 3.70, P-value = 0.0135), *Suttonella* (LDA score of 3.52, 0.0009) and *Janthinobacterium* (LDA score of 3.40, P-value = 0.0049).

### Microbiome function prediction analysis

**Fig. 7:**
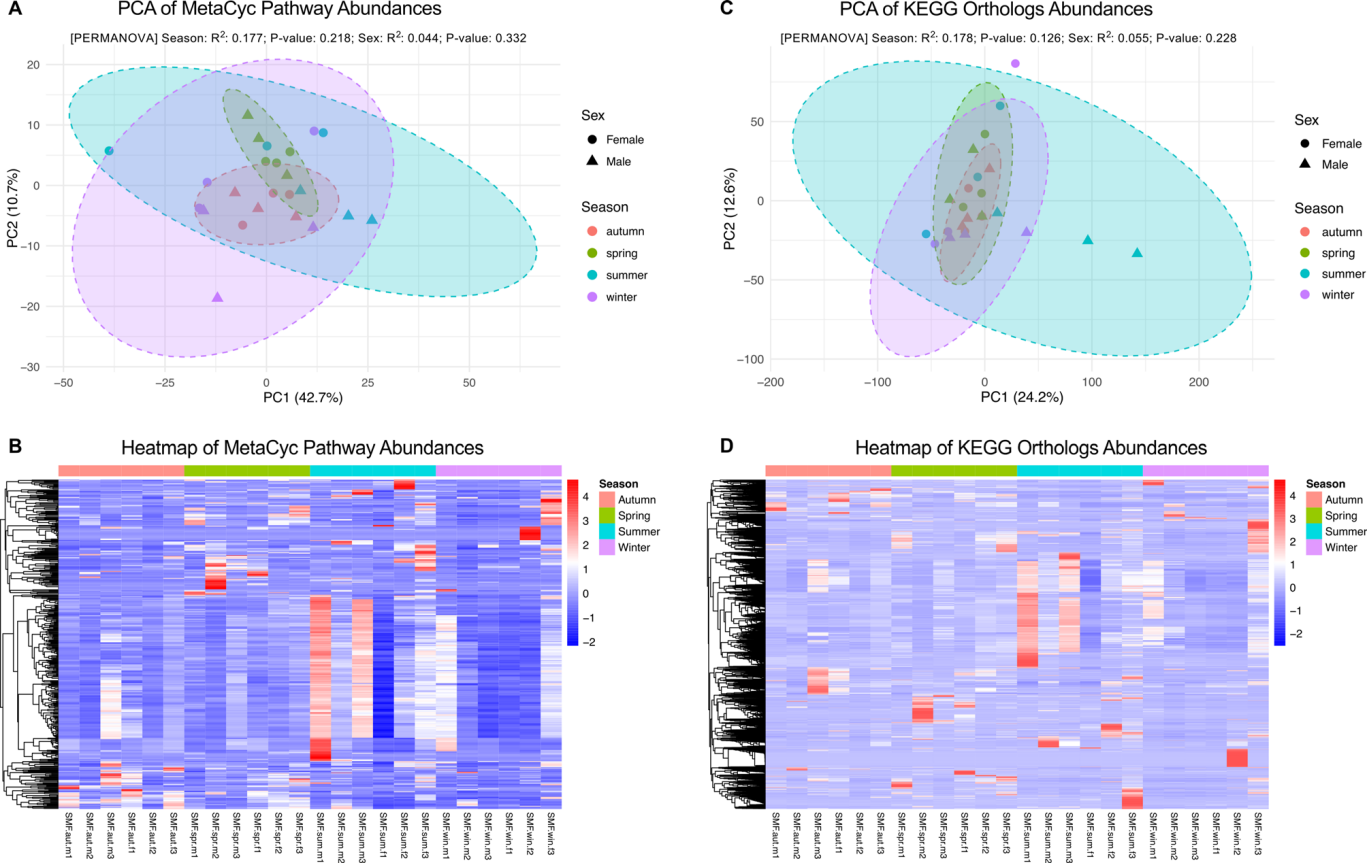
Functional predictions from 16 S rRNA gene data using PICRUSt2 across seasons and sexes. (**A**) PCA of MetaCyc pathway abundances showing sample clustering by “Season” (color) and “Sex” (shape). Ellipses represent 95% confidence intervals. PERMANOVA: Season: R² = 0.177, P-value = 0.001; Sex: R² = 0.044, P-value = 0.332. (**B**) Heatmap of MetaCyc pathway abundances. Rows are pathways and columns are samples, with color indicating normalized abundance (red = high, blue = low). Samples are annotated by “Season”. (**C**) PCA of KEGG Ortholog (KO)^38–40^ abundances, with clustering by “Season” and “Sex”. Ellipses represent 95% confidence intervals. PERMANOVA: Season: R² = 0.178, P-value = 0.001; Sex: R² = 0.055, P-value = 0.229. (**D**) Heatmap of KEGG Ortholog abundances. Rows are pathways and columns are samples, with color indicating normalized abundance (red = high, blue = low). Samples are annotated by “Season”.

To assess potential functional differences in the microbiome across experimental groups, a PICRUSt2 predictive metagenomic analysis based on 16 S rRNA gene data was performed, and PCA and Heatmaps were constructed based on abundances of MetaCyc pathways and, at the gene (or gene families) level, of KEGG Orthologs (KOs) (Fig. 7).

Principal Component Analysis of predicted MetaCyc pathway abundances revealed no distinct clustering, as groups confidence ellipses overlapped, suggesting the functional potential is broadly conserved across seasons. Moreover, PERMANOVA analysis did not detect statistically significant diferences between groups, although seasonal grouping explained a moderate portion of the variation (Season R² = 0.177, P-value = 0.218), while sex had a lower effect (Sex R² = 0.044, P-value = 0.332) (Fig. 7A). In accordance with these results, the heatmap revealed no season-specific nor sex-specific pattern in MetaCyc pathway abundance, and there was some variation within pools of flies from the same season (Fig. 7B).

**Fig. 8:**
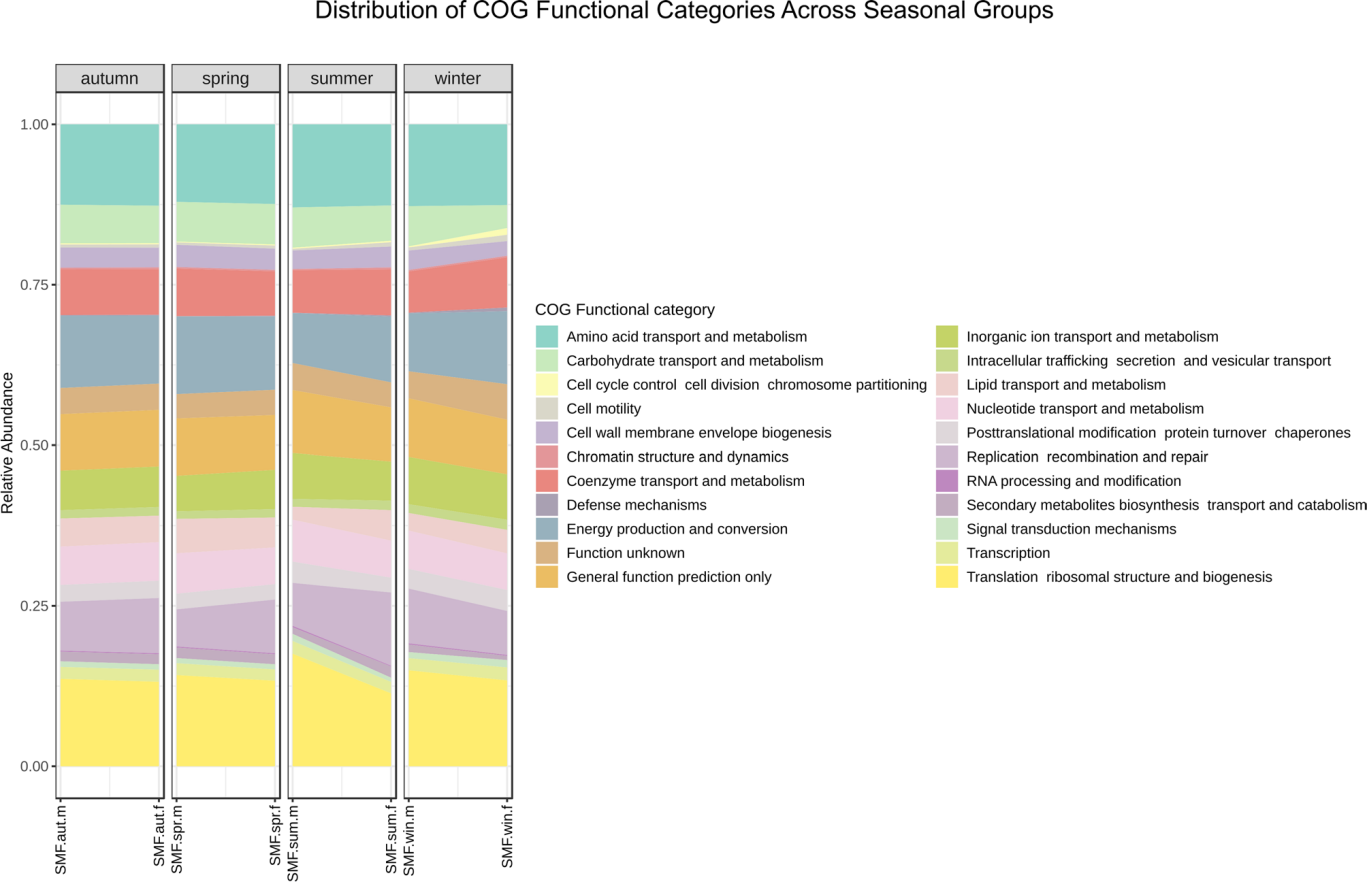
Functional composition of the microbiome based on predicted Clusters of Orthologous Groups (COG) categories, generated using Tax4Fun and the SILVA database. The graph illustrates the relative abundance of various COG functional categories, reflecting the potential metabolic capabilities of the microbiome within different seasons.

The PCA of KOs corroborated the lack of differences between pools of flies, as elipses overlapped and the PERMANOVA devolved an R^2^ of 0.178 (P-value = 0.126) for seasonal groups and an R^2^ of 0.055 (P-value = 0.228) for sex groups, meaning there were no significant differences in the overall functional gene profiles associated with either factor (Fig. 8C). In addition to this, the heatmap revealed that there were no group-specific clustering patterns, although there is sample-to-sample variation (Fig. 7D).

To complement this analysis, Tax4Fun was used to map the 16 S rRNA sequence data against the SILVA database and predict the abundance of specific Clusters of Orthologous Groups (COG) categories. This analysis provided a functional profile of the bacterial communities in seasonal groups (Fig. 8). Overall, functional profiling revealed minimal differences among groups despite the changes in taxa abundances, supporting the findings from the PICRUSt2 analysis.

## Discussion

SWD remains a huge threat to producers worldwide. It became a global pest due to its high environmental plasticity and the ability to survive for long periods in sub-optimal conditions such as those observed during the winter months in Mediterranean climates, and is still colonizing new territories. How the microbiome adjusts and contributes to this adaptation remains unknown. This study presents a comparative analysis of the bacterial community structure of different sex and seasonal wild SWD, using a 16 S metagenomics approach.

Overall, a stable community was identified, with phyla Proteobacteria and Firmicutes were dominant in all groups, mainly represented by the genera *Gluconobacter Pseudomonas*,* Commensalibacter* and *Pantoea*. These genera were also found throughout other SWD Portuguese populations, indicating that they are most probably “core” microbes. These genera are often featured on SWD gut 16 S metagenomic studies^9,42,43^ and laboratory isolations in culture media^19,21^. These data together suggest that a foundational bacterial community (a core) within SWD populations, independently of individual variation, might exist.

Besides having identified a stable community, our data supports high variability in function of environment and sex, in alignment with recent studies in *Drosophila* species^20,23,24^.

The high proportion of unassigned taxa in our dataset might reflect the combination of environmental exposure of wild *Drosophila* to diverse microbial communities, transient bacteria acquired from fruit substrates, and limitations in current reference databases, which are known to underrepresent many insect-associated microbial lineages. Similar patterns are often found in wild microbiome datasets, where unassigned taxa at the genus level can represent a high proportion of all taxa. No significant differences were observed regarding the number of observed ASVs (Chao1 index), either when comparing males and females or when comparing seasonal groups. However, SWD females, independently of season, present more diverse bacterial structures, with a more even distribution, comparatively to males, as they displayed significantly higher Shannon Index. These differences might be due to females having a higher foraging and locomotor activity than males^44,45^, and a higher nutritional demand (they require extra energy for oogenesis and finding suitable oviposition hosts)^5^, hence having a higher chance of harboring a more diverse microbiota.

Nonetheless, the Bray-Curtis PCoA analysis showed that the factor sex did not significantly influence the community composition, while season had a significant impact on the microbiomes. Moreover, the dendrograms showed that the winter and autumn groups tend to cluster together, in a different branch from the warm months groups, with the heatmap revealing similar bacterial abundance patterns within each season, evidencing once more a separation between seasonal groups. Because flies were collected from the same orchard across seasons, seasonal changes in host fruit availability and composition may have covaried with environmental conditions and thus also contributed to the observed beta-diversity patterns.

Seasonal shifts in microbiome composition have been reported in several insects, namely in *D. melanogaster*^46^, *Gryllus veletis*^29^ or *Apis melifera*^47^. These differences are likely influenced by host availability, as well as behavioral and physiological changes. We hypothesize that SWD winter individuals, which have limited access to preferred host such as fruits and nectar, and reduce foraging activity to conserve energy, may harbor a less diverse and distinct microbiota. In contrast, summer individuals engage actively in foraging and reproduction, hence increasing the chance of acquiring new/different microbes, comparatively to their cold months’ counterparts.

Seasonal differences in olfactory preferences and behavioral priorities further support this. In winter, SWD may shift their attraction from fresh fruit volatiles to fermentation cues^26^. Winter morphs also show down-regulation of metabolism-related genes and increased attraction to bitter compounds^48^. These adaptations are consistent with increased focus on sheltering and survival rather than feeding and could explain the higher bacterial diversity observed in summer, and the increased relative abundance of fermentation-associated bacteria such as *Acetobacter* and *Commensalibacter* in winter. Notably, *Commensalibacter* has been associated with increased winter survival in honey bees^49^, suggesting a potential beneficial role in cold tolerance.

Although the functional roles of many SWD-associated bacteria remain poorly understood, there is growing evidence that insect microbiota can influence thermal tolerance. In *D. subobscura* for instance, the gut microbiota improves heat tolerance^30^. Furthermore, *D. melanogaster* harboring disrupted microbiotas displayed altered cold tolerance^31^, being less prone to recover after a chill coma or acute cold exposure. Another study further disclosed that the gut microbiota might have a role in the transgenerational inheritance of low-temperature responses in *D. melanogaster*, as some cold-response genes were activated in control flies whose acquired microbiome had been previously exposed to cold^32^.

In SWD, a winter core microbiome within a two-year sampling period was identified^27^ and, even though our results show some bacteria are transient or present in significantly different abundances, we hypothesize they might be beneficial for winter adaptation and may re-integrate SWD microbiome in function of environment and diet.

While SWD winter diet remains unclear, studies suggest that decomposing organic matter such as rotten vegetables, fruits and leaves, as well as mushrooms, are alternative food sources during the cold months^27,50^. Another study demonstrated that SWD can even complete their life cycle in non-optimal diets, such as bird manure^28^, which is a source of early spring protein e.g. for the tephritid flies^51^. These studies indicate SWD might follow the “opportunistic” feeding pattern of other dipterans, which may shape its seasonal microbiota composition.

The LEfSe LDA analysis revealed significant (below the 10% threshold) enrichment of *Morganella*, *Methanosaeta*, *Serratia*, *Duganella*, *Frateuria*,* Suttonella and Janthinobacterium* in winter groups compared to other seasons.

Oxalobacteriaceae genera such as *Duganella* and *Janthinobacterium* are known for thriving in cold environments^52^, and Janthinobacterium has been found in winter morphotypes of SWD^27^. These bacteria may produce pigments (e.g. prodigiosin, violacein), with antifungal, antiprotozoal, and antiviral^53,54^. Thus, increased abundances of these genera could provide extra cold tolerance and indirect immune strategies to SWD.

The Enterobacteriaceae *Morganella* spp. were significantly more abundant in winter groups. Species from this genus are largely distributed in the environment and a gut commensal of humans, mammals, reptiles^55^, and even SWD^56^. In the tephritid fly, *Morganella* might be involved in nitrogen recycling, providing a metabolizable nitrogen source for the synthesis of essential amino acids, that can be reabsorbed by insect hosts^57^. In this sense, the increased *Morganella* abundance observed in winter groups could potentially help SWD in adaptation to low dietary nitrogen environments, such as during the winter when fruits are unattainable.

*Methanosaeta* (archaea) have a high affinity for acetic acid, and primarily use the aceticlastic pathway for methanogenesis. This genus is widely distributed and usually present in sludge/animal manure, as well as vegetable composts^58,59^ and can also be found in animals gut (e.g., animals with high rates of cellulose in their diet).

*Serratia* was also highly abundant in winter groups. Some *Serratia* species are known for regulating SWD genes encoding glycoside hydrolase, which participates in the digestion of plant polysaccharides (glucose, sucrose, starch) hence improving nutrient assimilation^14^, which could also contribute to SWD fitness during winter. Interestingly, there are also strains known for metabolizing organophosphate^60^ as well as carbamate insecticides^61^.

This is the first report of the presence of *Frateuria* spp. in SWD, although this genus was previously identified in insects, namely *F. defendens* in *Hyalesthes obsoletus*, the insect vector of Bois Boir^62^. *F. defendens* produced compounds with antimicrobial activity (e.g., 4-quinolinecarboxaldehyde and 5-hydroxymethyl-2-furaldehyde), and its effect was demonstrated against phytoplasma-related disease symptoms in grapevines^63^ and in mollycutes-related diseases^64^. It is possible that the production of these compounds might contribute to microbiome homeostasis and act as an indirect defense against bacteria that might be toxic to *Drosophila*. Other strains from this genus (e.g., *F. aurantia*) also have the ability to oxidize ethanol to acetic acid^65^. Together with, *Methanosaeta*,* Frateuria* strains should be expected in the microbiota of SWD with a diet that may be rich in acetic acid. Furthermore, some *Frateuria* species alongside with *Serratia*, have the ability to degrade toxic compounds produced by plants, e.g. terpenes and cyanates^66,67^.

*Suttonella* was also more abundant in winter and autumn groups. This genus includes only two formally described species: *S. indologenes*, linked to human respiratory infections, and *S. ornithocola*, associated with avian pneumonia^68^. Although rarely reported in microbiome studies, *Suttonella* and related taxa have been detected in the gastrointestinal tracts of animals such as rodents^69^. It is plausible that its seasonal enrichment in SWD may reflect environmental acquisition through coprophagy or contact with fecally contaminated organic matter.

Interestingly, the endosymbiont *Wolbachia* could also have a role in SWD cold survival as it was more abundant in cold months groups. Some *Wolbachia* strains are known to modulate the gut microbiota of *D. melanogaster*^70^, even though the prevalence and effects of *Wolbachia* on European SWD populations are still understudied. Nonetheless, *Wolbachia*-infected SWD displayed increased *Wolbachia* titers after cold exposure^71^. Moreover, *Wolbachia* can improve SWD performance after shifts in diet^72^, and is also known to protect SWD^73^ against viral infections. Interestingly, in crickets, *Wolbachia* increased its’ titer during overwinter, with its relative abundance returning to summer levels in spring^29^. Therefore, an increase in *Wolbachia* abundance in the cold seasons could possibly be linked to a, yet unclear, indirect mechanism of immune defense.

Despite observable changes in microbial community composition, the predicted MetaCyc pathway abundances, KEGG Ortholog (KO) profiles and Clusters of Orthologous Groups (COG) categories remained largely consistent. This functional redundancy suggests that SWD may maintain or selectively acquire microbial taxa that fulfill similar metabolic roles, thereby preserving core microbial functions regardless of taxonomic variation. Such stability in functional potential may reflect an ecological buffering mechanism, enabling the host to maintain essential microbiome-mediated processes despite environmental fluctuations and physiological differences. However, it is also possible that these microbiome shifts could be maladaptive under certain conditions. For instance, *Serratia* species are frequently reported as pathogens and may negatively impact SWD fitness^74^. Nonetheless, the increased abundance of these bacterial taxa in winter may contribute to host survival by enhancing cold tolerance, supplementing nutritional needs, detoxifying toxic plant compounds or pesticides, or offering protection against pathogens. A limitation of this study is that we did not include environmental controls, such as sequencing the bait or lure material used in the traps. The 8-hour trapping interval was selected for logistical reasons to ensure the collection of recently captured flies, but we cannot rule out the possibility that exposure to the trap environment or incidental contact with the bait may have contributed additional microbial signals. Future studies incorporating time-matched bait-only controls would help distinguish host-associated bacteria from potential environmental sources. Further work on microbe-microbe and microbe-host interactions will also be important to clarify the functional roles of these taxa.

In addition to this, it is important to note that fungi, especially fermentative yeasts, frequently form a key component of the microbiome in insects. For example, in natural-substrate studies of *Drosophila*, yeasts such as *Hanseniaspora uvarum* support larval growth during early stages of fruit fermentation, while bacteria dominate later in succession^75^. Fungal communities have also been shown to reflect the fruit substrate and persist through fly developmental stages in *D. suzukii*, implying their stable association and potential influence on microbiome assembly^76^. Given widespread evidence for bacterial–fungal interactions shaping microbial ecology, future studies should aim to integrate both bacterial and fungal profiling to provide a more complete picture of the insect microbiome.

In a general way, our data supports the existence of a core community independently of the environment, although the bacterial landscape was shaped by season, and less by sex. Our data supports the putative acquisition of beneficial bacteria during winter, although the overall microbiome function remained stable across groups. Nonetheless, these winter bacteria could improve SWD metabolism, cold-resistance and immunity, and future host-bacteria interaction studies should now focus on better understanding these relationships.

## Conclusions

The microbiome of SWD remains poorly characterized, despite the insect’s status as a significant and still growing global pest, with considerable economic impact.

The present study provides new insights into the composition, stability and potential functional roles of SWD-associated bacterial communities throughout seasons. This characterization is essential for understanding host-microbiome interactions and may offer perspectives on mechanisms underpinning SWD winter survival beyond phenotypical adaptations, with potential implications for the development of microbiome-informed biocontrol strategies.

This work tackled that characterization, and it identified core bacteria that remained associated within a Portuguese SWD population (as well as other populations within the same region), independently of season and sex, as well as taxa distinguishing seasonal groups. We discussed how enriched genera in winter morphotypes might assist SWD in winter survival, such as through cold protection, digestive enzymes, detoxification of allelochemicals and pesticides, or even confer protection against pathogens.

Predictive functional analyses revealed redundancy of microbial functions. However, putative roles, if any, of differently abundant genera in supporting growth and development and adaptation of the different SWD phenotypes, particularly winter SWD, remain to be determined. Future work could focus on experimentally studying the role of core and winter enriched bacteria in winter survival, for instance by disrupting winter microbiome or using different synthetic bacterial communities (“SynComs”) and assessing adult life span/survival under different diets and temperatures. From a biocontrol perspective, it would also be of interest to study if core bacteria and/or the seasonal shifts of SWD’s microbiome modulate the efficacy of biological control agents (BCA), potentially influencing the success of pest management programs.

## Supplementary Information

Below is the link to the electronic supplementary material.

Supplementary Material 1

## Data Availability

The datasets generated during and/or analyzed during the current study are available in the National Library of Medicine (NCBI) Sequence Read Archive (SRA) repository, [https://www.ncbi.nlm.nih.gov/sra/PRJNA1162420](https:/www.ncbi.nlm.nih.gov/sra/PRJNA1162420).
