# Supplementary material for: Seasonal dynamics and core stability of the bacterial microbiome of a Drosophila suzukii wild population
Source: Sci Rep. 2026 Jan 29;16:6569. doi: 10.1038/s41598-026-37656-y (PMC12909915; doi:10.1038/s41598-026-37656-y)
Supplement: Supplementary file 1 — Supplementary Material 1 [file 41598_2026_37656_MOESM1_ESM.docx]

**SUPPLEMENTARY DATA**

**Supplementary Table I.** – Sequencing data and quality of 16S rRNA sequencing. Raw reads is the original number of pair-ended reads; Raw tags is the number of combined tags from the pair-ended reads; Clean tags represents the number of tags after filtering and Effective Tags is the number of tags after chimera removal, which were used for further analysis. Average length is the average number of nucleotides of Effective Tags. Q20 and Q30 represent respectively the percentages of bases whose quality value in Effective Tags is greater that 20 (sequencing error rate is less than 1%) and 30 (sequencing error rate is less than 0.1%). GC (%) is the percentage of GC content in Effective Tags. Effective (%) is the percentage of no chimera Tags in Raw Reads.

| **SWD group** | **Raw reads** | **Raw Tags** | **Clean Tags** | **Effective Tags** | **Base(nt)** | **ASVs** | **Average Length(nt)** | **GC (%)** | **Q20 (%)** | **Q30 (%)** | **Effective Tags (%)** |
| --- | --- | --- | --- | --- | --- | --- | --- | --- | --- | --- | --- |
| Spring Males R1 | 133950 | 130917 | 127397 | 97734 | 41844806 | 316 | 428.15 | 52.92 | 97.47 | 92.61 | 72.96 |
| Spring Males R2 | 107571 | 106087 | 104459 | 79702 | 33029474 | 748 | 414.41 | 54.89 | 98.57 | 94.94 | 74.09 |
| Spring Males R3 | 131447 | 129049 | 127052 | 103940 | 43510348 | 498 | 418.61 | 53.09 | 98.23 | 94.09 | 79.07 |
| Spring Females R1 | 101374 | 98626 | 97330 | 77031 | 32333635 | 738 | 419.75 | 53.65 | 98.24 | 94.03 | 75.99 |
| Spring Females R2 | 133650 | 131615 | 129427 | 108866 | 46418195 | 579 | 426.38 | 54.30 | 98.17 | 93.95 | 81.46 |
| Spring Females R3 | 143811 | 140915 | 138096 | 104267 | 44517330 | 587 | 426.96 | 54.04 | 97.78 | 93.23 | 72.50 |
| Summer Males R1 | 134470 | 132756 | 130745 | 121634 | 51977116 | 514 | 427.32 | 55.73 | 98.26 | 94.09 | 90.45 |
| Summer Males R2 | 152473 | 129678 | 126718 | 97944 | 41130796 | 605 | 419.94 | 55.34 | 97.80 | 93.01 | 64.24 |
| Summer Males R3 | 133304 | 131682 | 129809 | 116525 | 49611579 | 457 | 425.76 | 55.52 | 98.35 | 94.35 | 87.41 |
| Summer Females R1 | 40456 | 38017 | 36896 | 32956 | 13282943 | 315 | 403.05 | 51.99 | 97.89 | 93.43 | 81.46 |
| Summer Females R2 | 123168 | 121640 | 119543 | 95464 | 39056210 | 322 | 409.12 | 53.76 | 98.57 | 94.99 | 77.51 |
| Aummer Females R3 | 132199 | 129953 | 128045 | 108169 | 45725511 | 811 | 422.72 | 52.03 | 98.19 | 93.98 | 81.82 |
| Autumn Males R1 | 136264 | 134123 | 132093 | 110584 | 46874330 | 349 | 423.88 | 52.99 | 98.16 | 94.01 | 81.15 |
| Autumn Males R2 | 134984 | 133253 | 131369 | 113077 | 48108291 | 299 | 425.45 | 54.00 | 98.22 | 93.90 | 83.77 |
| Autumn Males R3 | 137726 | 135589 | 133054 | 116222 | 47541761 | 636 | 409.06 | 52.80 | 98.41 | 94.69 | 84.39 |
| Autumn Females R1 | 131098 | 128934 | 126614 | 107985 | 44758219 | 566 | 414.49 | 51.72 | 98.31 | 94.32 | 82.37 |
| Autumn Females R2 | 133732 | 131898 | 129394 | 120162 | 50139398 | 272 | 417.27 | 54.90 | 98.48 | 94.77 | 89.85 |
| Autumn Females R3 | 129369 | 127274 | 124921 | 112653 | 47049094 | 379 | 417.65 | 54.01 | 98.35 | 94.51 | 87.08 |
| Winter Males R1 | 140608 | 138766 | 136396 | 107821 | 45491679 | 389 | 421.92 | 53.92 | 98.36 | 94.45 | 76.68 |
| Winter Males R2 | 163699 | 161598 | 157620 | 118033 | 50417762 | 230 | 427.15 | 53.69 | 97.86 | 93.24 | 72.10 |
| Winter Males R3 | 86020 | 84707 | 83224 | 72403 | 30150259 | 301 | 416.42 | 54.33% | 98.56% | 94.98% | 84.17% |
| Winter Females R1 | 98086 | 96946 | 95317 | 67226 | 27695747 | 374 | 411.98 | 52.91% | 98.54% | 94.89% | 68.54% |
| Winter Females R2 | 108165 | 106660 | 104765 | 81788 | 33105941 | 578 | 404.78 | 54.86% | 98.46% | 94.74% | 75.61% |
| Winter Females R3 | 133411 | 129691 | 126124 | 99222 | 42297446 | 455 | 426.29 | 53.02% | 97.89% | 93.38% | 74.37% |

**SUPPLEMENTARY FIGURE I.** – Heatmap showing the core microbiome composition of five different portuguese *Drosophila suzukii* populations. The plot displays bacterial genera detected at varying relative abundance thresholds (x-axis) and their prevalence across samples (color scale). Warmer colors represent higher prevalence, with red indicating genera found in all samples at a given abundance threshold. The core microbiome was defined as genera present in at least 50% of samples with a minimum relative abundance of 1%, highlighting the most consistently abundant taxa shared among samples.
